# The Effectiveness of Training Acceptance / Commitment and Training Emotion Regulation on High-Risk Behaviors of Students with Dyscalculia

**DOI:** 10.5812/ijhrba.10791

**Published:** 2013-09-20

**Authors:** Mohammad Narimani, Moslem Abbasi, Abbas Abolghasemi, Batoul Ahadi

**Affiliations:** 1 Department of Psychology, University of Mohaghegh Ardabili, Ardabil, IR Iran

**Keywords:** Acceptance, Commitment, Dyscalculia

## Abstract

**Background:**

Now a days the utilization of Acceptance / Commitment and Emotion Regulation Strategy as a comprehensive treatment plan has been discussed in both the prevention and the control of destructive and risky behaviors. Treatment based on Acceptance/Commitment and Emotion Regulation was effective in both the improvement and the control of high-risk behaviors of students with dyscalculia.

**Objectives:**

The purpose of this study was to investigate the effectiveness of Acceptance and Commitment, and Emotional Regulation training in high-risk behaviors of students with dyscalculia.

**Materials and Methods:**

This research was experimental, with pre-test, post-test and a control group. The statistical universe of this study included all sixth-grade male students in Ardabil city in the academic year of 2012-2013 (A.H.). The subjects of this study involved 800 sixth-grade elementary students in Ardabil province, selected using a multi-stage cluster sampling. From among them, 60 students with dyscalculia were selected using random sampling method after the initial diagnosis by structured clinical interview and the Keymath Mathematic test. Twenty pupil were selected for either the experimental or the control group. To collect data, the questionnaires of "Keymath Mathematic test" and High-risk Behavior" were used.

**Results:**

The results of Multivariate Analysis of Variance (MANOVA) showed that "Acceptance / Commitment and Emotion Regulation" treatment trainings were effective in reducing high-risk behaviors, in a manner that they led to a reduction in negative emotions, self-destructive and impulsive behaviors of students with math disorder (dyscalculia).

**Conclusions:**

It can be concluded that teaching these skills to the students has been influential in enhancing awareness level and change or positive attitude creation in the subjects. Therefore, it is essential to design and implement interventions based on "prevention caused by the peer group, in collaboration with the parents either at the school or at home among the family members".

## 1. Background

According to the definition of "Individuals with Disabilities Education Act" (IDEA), learning disability is a disorder in one or more basic of the psychological processes that involve understanding a language or its use. The disorder shows itself in the forms of disability in listening, thinking, speaking, reading and writing, spelling, and math calculations. However, it does not include those learning difficulties primarily resulting from visual, hearing or mobility disabilities, mental retardation, emotion disturbance, adverse environmental cultural or economic conditions (1).

Mathematical ability is as important as reading is important in the human life. However, researchers believe that the studies conducted regarding psychological processes, for math competency, or underlying failures of dyscalculia, have been much less than those conducted to study the inability to read. Math disability (dyscalculia) prevalence is estimated to be between 5% to 8% (1). Studies show that students with mathematics disabilities have some fundamental problems such as verbal problem solving and the related skills, diagnosis of obvious information in problems, using self-regulation and self-control approaches in homework performance processes, and maintaining their attention till the end of their homework. Learning disabilities cause difficulties for students in social, emotional and educational fields, where full understanding requires an attention to social, emotional and behavioral scopes of individual's life (2).

High-risk behaviors are among the psychological variables which show possibly due to the inability of the students in math learning. High-risk behavior is defined as behavior that results in increasing and devastating physical, psychological and social consequences to the individual. In adolescents these behaviors are strongly correlated with each other and follow the Co-variation Model. Presenting the term "Problematic Behavior Syndrome", ‎ Mohammadi and Abadi (3) believe that categories of high-risk behaviors include smoking, using drugs and alcohol, dangerous driving and early sexual activity. The results of a longitudinal study showed that from among 22 children diagnosed with learning disabilities in childhood, about 27% were delinquency cases aged between 17 and 18, and less than 10% had criminal records (3).

Among the treatments that can could reduce high-risk behaviors of students with math learning disabilities effects, while they have not been considered by researchers, we could refer to Acceptance and Commitment training as well as Emotional training. Accepting an important alternative to avoid in accordance with experience includes active and conscious acceptance of personal events related to the history of an individual. In fact, the main goal of this treatment is making psychological flexibility; meaning to create the capability of practical selection from among different choices which is more suitable (4). 

Considering the novelty of this treatment method, no research has examined its efficacy directly on improving the problems of students with dyscalculia. However, regarding the efficacy of this treatment on reducing social-psychological problems, along with the fact that students with learning disabilities are also involved with these troubles, it is expected that this drug could be effective in reducing the problems of this group of disorders. Empirical evidences about the efficacy of therapy based on Acceptance and Commitment in disorders like psychosis (4), risky behaviors such as alcohol and drug abuse, depression and social phobia have been identified and determined. 

Emotion regulation is among the trainings that its educational aspects has less been used on children with learning disabilities, especially those with dyscalculia (5). Emotion regulation refers to the ability to understand the emotions, modulating experience and expressing emotions (6). Adaptive emotion regulation is associated with adaption, psychological well-being, and positive social interactions. The study of psychological studies and literature review indicate that emotion regulation is an important factor in determining the health and having good performance in social interactions and reducing the risky behaviors. A little research has been done in terms of interventions based on emotion regulation. 

A research conducted by Narimani and Rajabi (7) showed that antisocial and aggressive behaviors of students with learning disabilities were significantly more than ordinary students. Ghosh (8) in a study found that the rates of behavioral problems in students with learning disabilities were higher than that of ordinary students. These students have difficulty in starting and sustaining friendships, and these problems may lead to loneliness, low self-esteem, depression and other psychological-social problems. Lee and Tsang (9) showed in a research that cognition and regulation of emotions and training social problem-solving with cognitive style improves significantly the performance of students with learning disabilities in terms of increasing social problem-solving, decreasing undesirable behaviors, aggression, isolation and also changing societal goals. Moreover, Hayes (10) in his research entitled "Acceptance and Commitment and their Role in Adaptation, Social-psychological Well-being of Children with Behavior Problems" showed that Acceptance and Commitment were associated with to social-psychological well-being and improvement of social relationships of children with behavior problems, and better adaptation of them with relevant community. This issue is associated with high level of awareness.

As indicated in various researches, Acceptance and Commitment training, in which mindfulness was its essential component had been effective on variables such as anxiety, depression, stress, health, adaptation and reduction of behavioral problems in children. Solaman (11) showed in his investigations that cognitive interventions was effective in increasing the understanding of social-emotional behaviors and difficult children behaviors modification with learning disabilities. And these interventions were the cause of increasing students' thinking power concerning making theory and understanding humor in social relationships.

In general, bearing in mind the long-term consequences of mathematical disorder (dyscalculia) and its increasing prevalence among school students and fundamental role of mathematics in modern life, proper planning in their rehabilitation and remediating their learning problems is necessary. In addition, if the disorder is diagnosed and treated in elementary schools, it would be much less problems in adulthood. Otherwise, the stricken individual may suffer from different problems in adulthood including compatibility, emotional and social issues (10, 11). 

Most significantly, considering different emotional problems of this group of students, using given methods in this respect is of high importance. Also, performing few studies on this issue and the lack of research on the effectiveness of these approaches on the emotional and social problems of students with learning disability, using the results of this research in treatment, counseling and providing basis for further researches are among the reasons for doing this study. Hence, in this study we are seeking to answer the question: "Are Acceptance, Commitment and Emotion Regulation therapies effective in reducing risky behaviors of students with math learning disabilities (dyscalculia)?"

## 2. Objectives

The purpose of this study was to investigate the effectiveness of Acceptance and Commitment, and Emotional Regulation training on high-risk behaviors of students with dyscalculia. 

## 3. Materials and Methods

This research is experimental pre-test and post-test study with a control group. In this study, treatment methods at three levels of treatment training based on Acceptance and Commitment, Emotion Regulation and lack of training (the control group) were considered as active independent variable, and high-risk behaviors as dependent variables. The statistical universe of the study included all sixth grade elementary male students in Ardabil city in the academic year 2012-13 (A.D). The study sample consisted of 800 sixth-grade students in Ardabil province, selected through a multi-stage cluster sampling from among the sixth-grade elementary school male students in Ardabil city. These students were evaluated by key-math mathematics test and the cases suspected to mathematical disorders (dyscalculia) were being interviewed clinically in a structured way. Finally, 60 cases were simply and randomly selected from among the students having math problems. Despite the fact that in experimental investigations the minimum number of sample should be 15 people (12), in this research 60 subjects with mathematical problems (for each sub-group, n = 20) was selected as the research sample with the aim of increasing external validity of the study. The inclusion criteria for this study are as follow: 1-The sixth-grade elementary student’s 2-Male Sex 3-Lacking comorbid behavioral disorders 4-Educated in the public schools. To gather the data in the present study, we have used the following tools:

### 3.1. Structured Clinical Interview for DSM-IV Disorders

SCID is a semi-structured clinical interview being used for the diagnosis of axis I disorder according to the DSM. In a study conducted by Beskow et al. SCID potential benefits were tested for using in mental health clinic. The study concluded that SCID could be used to ensure a reliable and accurate diagnosis (13).

### 3.2. Key-math Mathematics Test

Key-math Mathematics Test has been normalized by Kernolie, Natchiman and Prietchet in 1976. The test is used to determine strengths and weaknesses in students' knowledge in different fields of mathematics. Reliability coefficient of this test was obtained as 0.80 using Cronbach's alpha (14). This test has been used in order to identify students with dyscalculia (mathematics disorder). 

### 3.3. High-risk Behavior Questionnaire

In order to determine the prevalence rate and identify protective-incentive factors of risky behaviors, in the present study we used Mohammadi's high-risk behavior questionnaire (3). This questionnaire has 38 questions. Each one is graded on the basis of the 5-degree Likert scale ("totally disagree" to "totally agree"). The scale includes seven components (tendency to narcotics, tendency to alcohol, tendency to violence, tendency to relationship and sexual behavior, tendency to heterosexual relationship, and tendency to dangerous driving). Psychometric properties in the standardized version of Mohammadi are promising concerning the difference diagnosis among the groups. Face validity of the questionnaire has been reported as equal to 0.77 by Rajabi and Cronbach's alpha‎ coefficient as 0.67.

### 3.4. Implementation Method

After coordination and permission, Key-Math mathematics test was completed by the study’s sample students. The subjects with high scores were identified and clinically interviewed. Then the students with math learning disabilities (dyscalculia) were randomly assigned to two experimental groups and one control group. Justifying the subjects and expressing the objectives, the subjects were requested to participate in the treatment course of this disorder. 

Before starting the training methods, all three groups under study were be pre-tested and were asked to complete the predetermined questionnaires. The duration of therapy sessions in each treatment method was eight sessions, each lasting for one and a half hour. It was performed in groups once a week, in an area determined by the Department of Ardabil City Education. After completing the course of training, the treatment and control group were post-tested. 

Finally, the collected data was analyzed by multivariate analysis of variance (MANOVA). In addition, assurances about information confidentiality and freedom of choice for taking part in the study were among the ethical principles which driven the research.

### 3.5. Intervention Methods

The intervention method has shown in the form of a Table 1 below. 

**Table 1.** Intervention Methods

Treatment Based on Acceptance and Commitment	
**First Session**	
Conducting Pre-test, acquainting group members with each other, with therapist, and general treatment plan and the sessions.	
**Emotion Regulation Training**	
**First Session**	
Conducting Pre-test, communication and conceptualization.	
**Second and Third Sessions**	
Understanding the therapeutic concepts of ACT (mental flexibility, psychological acceptance, emotional awareness, cognitive isolation, self-image, personal stories, values clarification and acting with responsibility)	
**Emotion Regulation Training**	
**Second Session**	
Review of the previous session; Training "awareness of positive emotions" (happiness, interest and love ), paying attention to the positive emotions, and the need to use them along with the examples in the form of visualization, home assignments, writing positive main emotions and registering them in the associated form.	
**Third Session**	
Review of the previous session; Training "awareness of negative emotions" (Anxiety, sadness, anger and hatred), paying attention to the negative emotions, and the need to use them along with the examples in the form of visualization, home assignments, writing negative main emotions and registering them in the associated form.	
**Fourth and Fifth Sessions**	
Focusing on enhancing mental awareness and training accountability and appropriate exposure with the subjective experience, defining target and attempting to achieve it.	
**Emotion Regulation Training**	
**Fourth Session**	
Review of the previous session; Teaching to accept and embrace positive emotions and accept positive emotions without judgment and its positive and negative effects, homework assignments and surveying parents and close friends regarding low or high levels of positive emotions, and registering them in the associated form.	
**Fifth Session**	
Teaching the fourth session, but for negative emotions along with home assignment of the same session regarding negative emotions.	
**Sixth and Seventh Sessions**
Using the learnt, providing feedback by the group and the therapist
**Emotion Regulation Training**
**Sixth Session**
Review of the previous session; Training of re-evaluation and expressing positive emotions, Training of subjective experience of positive emotions in the form of visualization (happiness, interest and love), mental inhibition, and training suitable expression of these emotions
**Seventh Session**
Training of re-evaluation and expressing negative emotions, Review of the previous session, Training of subjective experience of negative emotions (anxiety, sadness, anger and hatred), inappropriate expression, and inhibiting inappropriate expression of these emotions.
**Eighth Session**
Conclusion and conducting post-test
**Emotion Regulation Training**
**Eighth Session**
Concluding the educational sessions and conducting post-test.

## 4. Results

Mean and age standard deviation (Mean ± SD) of students with math problems (dyscalculia) in the test and control groups were (12.28 ± 1.63), and (11.67 ± 1.35), respectively.

**Table 2.** The Mean and Standard Deviation of High-Risk Components in Acceptance and Commitment Training Pre-Test and Post-Test and Emotion Regulation and Control Group

	Acceptance and Commitment, Mean ± SD		Emotion Regulation, Mean ± SD		Control Group, Mean ± SD	
**Tendency to narcotics**						
Pre-test	23.95 ± 3.4		24.17 ± 3.14		24.17 ± 3.12	
Post-test	21.14 ± 2.89		21.43 ± 3.01		23.17 ± 3.14	
**Tendency to alcohol**						
Pre-test	22.32 ± 2.88		21.69 ± 2.54		22.19 ± 2.45	
Post-test	17.87 ± 2.34		16.32 ± 2.34		21.34 ± 2.23	
**Tendency to cigarette smoking**						
Pre-test	17.12 ± 1.89		17.1 ± 1.65		18.9 ± 2.08	
Post-test	14.32 ± 1.54		14.52 ± 1.23		17.46 ± 2.03	
**Tendency to violence**						
Pre-test	18.38 ± 2.3		19.49 ± 2.03		19.32 ± 2.65	
Post-test	14.67 ± 1.87		13.68 ± 1.89		18.65 ± 2.56	
**Dangerous relationship and sexual behavior**						
Pre-test	13.19 ± 1.17		13.65 ± 1.22		14.38 ± 1.87	
Post-test	9.48 ± 1.14		10.69 ± 1.13		13.76 ± 1.68	
**Tendency to heterosexual relationship**						
Pre-test	14.73 ± 2.1		13.87 ± 1.62		15.65 ± 1.49	
Post-test	11.55 ± 2		10.86 ± 1.49		14.32 ± 1.33	
**Tendency to dangerous driving**						
Pre-test	17.65 ± 2.12		16.43 ± 2.03		16.76 ± 1.88	
Post-test	14.33 ± 1.97		13.51 ± 1.87		15.67 ± 1.76	
**Total score**						
Pre-test	115.87 ± 12.32		117.34 ± 13.45		119.67 ± 13.89	
Post-test	105.18 ± 10.68		106.17 ± 10.87		111.96 ± 11.05	

As it can be seen in Table 2, (Mean and standard deviation) of pre-test total score of high-risk behaviors in students with math problems (dyscalculia) in educational department of treatment based on Acceptance and Commitment, the Emotion Regulation and control group therapy are equal to (115.87 ± 12.32), (117.34 ± 13.45), and (119.67 ± 13.89), respectively. 

The results of Wilks’ Lambda test indicated that the group effects on the composition of high-risk behaviors’ components was significant error [Wilks, F (12, 543) = 0.124, P < 0.001]. 

The above test could allow the use of multivariate analysis of variance (MANOVA). The results showed that there was a significant difference at least between one of the investigated variables existed in the two studied groups. Before using parametric test of covariance analysis to meet its assumptions, the Box’s and Levene’s tests were used. According to the Box’s test which has not been significant for any of the variables, the condition of variance/covariance matrix homogeneity has properly been observed (Box = 19.745, F = 2.14, P = 0.34).

**Table 3.** Results Of Multivariate Analysis Of Variance (MANOVA) To Compare The Difference Between The Pre-Test And Post-Test Of High-Risk Behaviors Component Scores In Three Experimental Groups Of Training "Acceptance/Commitment", Emotion Regulation Training And "Control Group"

Dependent Variable	SS^*^	df^*^	MS^*^	F	P
**Tendency to narcotics**	131.49	2	131.49	11.67	0.001
**Tendency to alcohol**	112.45	2	112.45	9.231	0.001
**Tendency to violence**	231.23	2	231.23	13.23	0.001
**Tendency to relationship and sexual behavior**	203.46	2	203.46	12.42	0.001
**Tendency to heterosexual relationship**	108.72	2	108.72	8.98	0.001
**Tendency to dangerous driving**	198.33	2	198.33	6.212	0.001
**High-risk behaviors**	276.98	2	276.98	11.32	0.001

^*^Abbreviations: SS, sum of squares; MS, mean square; df: Degree of Freedom

The results of Table 3 show that there is a significant difference between the mean scores of tendency to narcotics (F = 11.67), tendency to alcohol (F = 9.231), tendency to violence (F = 13.23), tendency to relationship and sexual behavior (F = 12.42), tendency to heterosexual relationship (F = 8.98), and tendency to dangerous driving (F = 6.212) in experimental groups of training “Acceptance and Commitment” and “Control Group” (R < 0.001). In other words, the training of Acceptance/Commitment and Emotion Regulation Training therapy has reduced the components of high-risk behaviors. 

## 5. Discussion

The purpose of the present study was to determine the effectiveness of Acceptance and Commitment trainings and Emotion Regulation on high-risk behaviors of students with dyscalculia (math learning disability).

The results showed that treatment based on Acceptance and Commitment is effective on the improvement and control of high-risk behaviors of students with dyscalculia. Compared with the control group who were on a waiting list, the experimental group who received treatment based on acceptance and commitment, showed a more consistent and more significant improvement in the dependent variable. The results of this study are consistent with other research findings (15).

In explaining these findings, we can say that "acceptance", which is often considered a form of mindfulness, is awareness of each thought, simply and without details, in the absence of judgment, prejudice, and present-oriented. In this way, thoughts, feelings and sensations are accepted as they are (16).

In fact, the treatment program teaches patients to reevaluate behaviors those do not directly threaten life, but menace the sound quality of life and tries to find flexible solutions to apply some changes in these conditions. This is a chance and an opportunity for the patients to receive the first stage of therapy with a commitment to change. For instance, among the behaviors that threaten the quality of life we can refer to the following:

Excessive impulsivity, fearless driving, unrestrained sexuality, keeping on destructive and dysfunctional interpersonal behaviors, high-risk behaviors, substance abuse, and interpersonal problems associated with lack of emotional control and leads to behaviors which menace the individual quality of life level. During the treatment, the therapist instructs the patient about traumatic properties of these behaviors. She/he explains the reason why the treatment invading behaviors have to be stopped. 

Therefore, in dealing with such behaviors, foremost the patient must be committed to changing herself/himself. Moreover, she/he should accept that she/he holds these behaviors and only she/he has the ability to change them. The patient and therapist discuss the issue or problem and focus on the solution to experience new emotions. This treatment gives a person the second opportunity to view, describe and explain emotional states without any judgment.

 In this way, the therapist is mostly focused on guiding the patient towards the full consciousness and the patient takes the responsibility. 

The therapist encourages the patient to experience perfectly the thoughts and emotions associated with a particular thinking, feeling or behavior, without suppressing them, making value judgments about, and experience the secondary emotions such as shame and guilt after experiencing this behavior, thought and emotion (17).

The results also showed that students who had received treatment based on emotion regulation, compared with their counterparts in the control group not receiving any treatment could better control their emotions. These results are consistent with other research findings (18). 

Empirical researches conducted in this area have shown that people with thrill-seeking behavior, impulsivity and risky behaviors were more successful to control their ruminative thoughts, tempting narcotics, aggression and anxiety when they had participated in a treatment course of emotion regulation skills.

Therefore, using emotion regulation strategies at present as a comprehensive treatment plan has been discussed both in the prevention and control of destructive and risky behaviors. To explain these findings, one could say that the difficulty in self-regulation of emotions or inability to cognitive processing of emotion regulation or proper revealing of emotions (19), in other words, lack of perception possibility and evaluation of emotional information in cognitive processes confuse and frustrate the individual emotionally and cognitively.

On the other hand, the empirical evidences show that parents having close relationships with children create more positive emotions in them (20). These emotions (excitement) influence the variables of self-esteem and self-concept and this inhibits the formation of negative emotions in unfortunate situations.

On the other hand, when parents do not express their emotions properly, and not only do not support their child in situations where he/she fails, but also make use of contempt and reproach, the child will not be able to identify the emotions properly. This is called Alexithymia in psychology. They only see themselves in situations, and relate their success to the situations, and not to their capabilities. Moreover they relate their failures to individual factors.

In explaining these findings, we can say that "parenting style", which is associated with high control or rejection, leads to the development of anxiety and depression in children through the growth of non-functional cognitive schemas that are biased towards the risk and negative consequences (21).

This causes the individual to turn to high-risk and inappropriate behaviors in order to get rid of these emotions and reset them, since substance abuse, alcohol and intrapersonal difficulties because a person to be freed momentarily and there will be a world of calm and receptive. In a practical point of view, this research could have bilateral effects on family psychology as well as educational psychology. 

The findings of this study could aid Education and Higher Education officials to improve learning problems and impulsive behaviors of these students. Considering the role of family and parenting processes in positive emotions, negative emotions and emotion regulation of these students, the necessity of holding special training courses for parents and the necessity of creating closer relationship between family and school to optimize the performance of students in mathematics in education system is very important.

The study is about Ardabil city students, and the studied sample includes elementary school male students. Therefore, the generalizability of the results is limited. Lack of follow-up, the time limit for providing education, and lack of control associated with pre-test effects are among the other limitations of the present study. It is suggested that future researches should consider these issues.

## References

[1] Forrester JE, Tucker KL, Gorbach SL (2004). Dietary intake and body mass index in HIV-positive and HIV-negative drug abusers of Hispanic ethnicity.. Public Health Nutr..

[2] Shakibi MR (2004). Prevalence of Opium Addiction in Iranian Drivers 2001-2003.. J Med Sci..

[3] Zadeh Mohammadi A, Ahmad Abadi Z (2006). Simultaneous Occurrence Of High-Risk Behaviors Among High School Adolescents In Tehran.. J Fam Res..

[4] Saeland M, Haugen M, Eriksen FL, Smehaugen A, Wandel M, Bohmer T (2009). Living as a drug addict in Oslo, Norway--a study focusing on nutrition and health.. Public Health Nutr..

[5] Baptist J, Minnie L, Buksner S, Kaye R, Morgan J (2007). Screening in the early years of mathematics: Identifying red flag to support early learners at risk.. Orbit..

[6] Gross JJ (2002). Emotion regulation: affective, cognitive, and social consequences.. Psychophysiology..

[7] NarimaniI M, Rajabi S (2006). A study of the prevalence and causes of learning disorders among elementary students of Ardabil province.. Res Except Child..

[8] Ghosh A (2007). Comparison of anthropometric, metabolic and dietary fatty acids profiles in lean and obese dyslipidaemic Asian Indian male subjects.. Eur J Clin Nutr..

[9] Lee A, Tsang CK (2004). Youth risk behaviour in a Chinese population: a territory-wide youth risk behavioural surveillance in Hong Kong.. Public Health..

[10] Hayes SC (2004). Acceptance and commitment therapy, relational frame theory, and the third wave of behavioral and cognitive therapies.. J Behav Ther..

[11] (2006). Risky behaviors and prevalence at aids in adolescents..

[12] Delavar A (2011). Theoretical And Practical Research Foundations In The Humanities And Social Sciences..

[13] Mohammad Khani P, Jahani A, Tamana'ifar S (2005). Structured Clinical Interview For DSM Disorders..

[14] Esma’il M, Heydarali E, Heydarali H (2001). Adaptation And Normalization Of Key-Math Mathematics Test..

[15] Shareh H (2008). Demographic Variable Associated With TeenageGirls’ Risk for Running Away From Home in Mashhad.. J Found Ment Health..

[16] Divesalar K, Nakhaee N (2007). Smoking Prevalence and Its Related Factors in Students of Two Universities in Kerman.. J Baboul Un Med Sci..

[17] Toprak S, Cetin I, Guven T, Can G, Demircan C (2011). Self-harm, suicidal ideation and suicide attempts among college students.. Psychiatry Res..

[18] Toprak S, Cetin I, Akgul E, Can G (2010). Factors associated with illicit drug abuse among Turkish college students.. J Addict Med..

[19] Serras A, Saules KK, Cranford JA, Eisenberg D (2010). Self-injury, substance use, and associated risk factors in a multi-campus probability sample of college students.. Psychol Addict Behav..

[20] Hemati J, Eslami S, Norbakhsh M (2007). Comparing the Effects of Social- Mental Stress Factors on Athlete Girl and Boy Students’Mental Health With Non-Athlete Ones.. J harkat..

[21] Hossaieni H, Kazemi S, Shahbaznejad L (2006). The Relationship Between Exercise and Mental Health of Students.. J Mazandaran Un Med Sci..

